# Age differences in diabetes-related complications and glycemic control

**DOI:** 10.1186/s12902-017-0175-5

**Published:** 2017-05-04

**Authors:** S. M. Shamshirgaran, A. Mamaghanian, A. Aliasgarzadeh, N. Aiminisani, M. Iranparvar-Alamdari, J. Ataie

**Affiliations:** 1 Department of Statistics and Epidemiology, School of Health Sciences Tabriz University of Medical Sciences, Tabriz, Iran; 2 Injury Epidemiology Prevention Research Center, Tabriz University of Medical Sciences, Tabriz, Iran; 3 Endocrine Research Center, Tabriz University of Medical Sciences, Tabriz, Iran; 4 Department of Internal Medicine, School of Medicine, Ardabil University of Medical Sciences, Ardabil, Iran; 5 Khalkhal Health Center, School of Khalkhal Medical Sciences, Ardabil University of Medical Sciences, Ardabil, Iran

**Keywords:** Diabetes mellitus, Hyperglycemia, Complication, Comorbidity, Blood glucose

## Abstract

**Background:**

This study aimed to examine the associations of age with the presence of complications and glycemic control in the Northwest of Iran.

**Methods:**

A total of 649 people with diabetes who were >25 years old and had a caring record in diabetes clinics in two Northwestern provinces of Iran during 2014–15, were recruited in this cross-sectional study. General information including demographic, socioeconomic status and lifestyle factors were collected by trained interviewers. Clinical information was retrieved from clinic's record. Univariate and multivariate logistic regression were performed to assess the predictors of diabetes outcome of interest as well as to clarify the role of age in relation to these outcomes.

**Results:**

Compared to the age group of ≤49, the middle age group (50–59) and the older age group (60 years of age and older) were less likely to report poor glycemic control (OR fully adjusted = 0.49 95% CI: 0.28–0.86 and (OR = 0.44 95% CI: 0.24–0.80), respectively. Additionally, poor glycemic control was associated with income level, disease duration, hypercholesterolemia, high level of LDL and hypertension. In contrast, age was associated with the highest percentage of complications. People with duration of >7 years of disease record were 6 times more likely to have complications (OR_adj_ = 5.98 95% CI: 2.35–15.22).

**Conclusion:**

Although the prevalence of complications was higher among the older diabetic patients, they had a better glycemic control. The influential factors were variably associated with the two diabetes-related outcomes; therefore, a more comprehensive risk profiles assessment is needed for glycemic control.

**Electronic supplementary material:**

The online version of this article (doi:10.1186/s12902-017-0175-5) contains supplementary material, which is available to authorized users.

## Background

Diabetes is a global concern driven by both population growth and ageing [1]. There were 387 million people with diabetes in 2013 and that is predicted to reach 592 million by 2035. The majority of these people fall between the ages of 40 and 59, and 80% of them live in low- and middle-income countries [2].

Diabetes as a chronic disease has a negative impact on different organs due to the negative effect of hyperglycemia and requires ongoing management. There is evidence that diabetic complications, hospitalization and mortality are more prevalent among older people with diabetes [3–6]. In contrast, the relationship between age and glycemic control has been reported to be inconclusive. Some studies have reported a high prevalence of poor control in the elderly [7], there are studies which point to no effect of age on metabolic control [8], to better glycemic control among older people [9–12] and even point to an inverse relationship between age and glycemic control [13, 14]. Worse glycemic control and lipid profile, higher prevalence of obesity and additional cardiovascular risk factors, remarkable increase in the risk of cardiovascular disease, higher rate of death from hyperglycemic crisis, among young diabetic type 2 patients were reported by some studies [15–18]. Recent trend analysis of diabetic complications in the USA showed a decline in the end- stage renal disease across all age groups from 2000 to 2010, however the age difference for rates of acute myocardial infarction and stroke has been narrowed, bring this to the attention that younger adults with diabetes require more rigorous care and management [19].

Better glycemic control among older people might be explained by other factors such as Body Mass Index (BMI) and disease duration [20, 21]. However, some studies found an independent role for age after consideration of BMI and disease duration [9, 13]. Therefore, the aim of this study was to examine age-related associations with the presence of complications and glycemic control in people with type 2 diabetes.

We conducted this study in the northwest of Iran: Ardabil and East Azerbaijan provinces. Almost all the people living in these areas have Azari-Turkish background with different sociocultural values influencing their lifestyle. Diabetes is a major concern in these areas [22, 23] where not only have previous studies shown inadequate diabetes care and high prevalence of complications but relatively poor quality of life among these people has been referred to as well [24–27].

## Methods

A cross-sectional study was conducted in the Northwest of Iran. People with T2DM referring to diabetes clinics (Imam Khomeini Hospital in Ardabil during January and May 2014, and Imam Reza and Sina in Tabriz during November 2014 to March 2015) were selected through a convenience sampling method. These centers are referral clinics in these areas in the Northwest of Iran. The study inclusion criteria were: diagnosis of T2DM, age ≥25 years of old, having a caring record in the clinic, at least, for one year, residing urban regions of the provinces, and not having special and debilitating diseases (hemophilia and thalassemia). Exclusion criteria were unwillingness to participate in the study and having other types of diabetes.

The present study was approved by the Ethics Committee of Tabriz University of Medical Sciences (Ethic numbers TBZMED.REC.1392.207 and TBZMED.REC.1394.55), and at the beginning of the study, informed consent was obtained in written forms from all of the participants.

### Measurements

Data was collected using a structured questionnaire by two trained interviewers. The questionnaire was divided into two parts. Part one consists of general information including age, gender, place of residence, marital status, monthly household income, health insurance, education level, weight and height. Part two includes clinical features as such blood pressure, lipid profile, disease duration. Moreover, complications i.e. neuropathy, retinopathy, nephropathy and cardiovascular complications were gathered (Additional file 1). Age was classified into 3 categories of ≤49, 50–59 and ≥60 years of age. For the purpose of some analysis, it was integrated into two categories of <60, ≥60 years old. Some predictors were drawn from clinic's records such as blood pressure, BMI, total cholesterol, Fasting Blood Sugar (FBS) and were defined as: controlled hypertension (systolic blood pressure <140 mmHg, and diastolic <90 mmHg), and desirable cholesterol <240 mg/dl, HbA1C <7%, and FBS < 126 mg/dl, based on the recent American Diabetes Association recommendation [28]. BMI was classed into three categories; <25, 25–29.9, and ≥30.

### Statistical analysis

Descriptive analyses were used to describe the general characteristics of the study population. Two diabetes care outcomes, that is presence of any complication and glycemic control, based on HbA1C values, were chosen and described based on a range of predictors of interest. Then a univariate analysis was performed to assess the association between age and a list of covariates. Multivariable logistic regression models with glycemic control and presence of complications as the dependent variable were used to estimate odds ratios (ORs) with 95% confidence intervals (CI). Two models were built: adjusted for age, sex, and measures of socioeconomic status (educational attainment and income); and adjusted for age, sex, measures of socioeconomic status and related clinical factors (BMI, lipid profile, hypertension, and duration of disease). In order to analyze the independent association of the age factor with glycemic control and presence of complications, the basic analysis was repeated, separately, with adjustment for each influential predictor. Analysis was performed using the statistical package for social science (SPSS) Version 23 and a significance level of 5% was set prior to the initiation of the study.

## Results

A total of 694 people with T2DM were included in this study. The mean age of the participants was 55.71 (8.99), about 70% of them were female, 89% married, 47% illiterate, 20% of them were engaged in paid work, and the monthly income of 63.3% were reported to be less than 1000,000 Tomans,^1^ less than 2% of the participants were smokers, and the majority of them (80%) was overweight/obese. Approximately, duration of disease among 58.6% of the patients was more than 7 years; about 50% had, at least, one complication. 37.5% of participants in the old age group (≥60) were male whilst it was 26.8% in younger age group (*P* < 0.001). Higher percentages of older participants (59.2%) were illiterate and 75.7% of them had low level of income which was statistically significant (*P* < 0.001 & *P* = 0.003 respectively). The prevalence of hypertension was significantly higher among older age group than younger adults (63.1% vs. 53.7%, *P* = 0.019). Moreover, complications were higher among elderly compare to younger participants (57.1% vs. 45.2%. *P* = 0.003) (Table 1). The frequency of complications in this study according to the patient’s clinic record were neuropathy (34.9%), retinopathy (16%), nephropathy (5.9%), stroke (1.6%) and cardiovascular disease (6.1%).

**Table 1:** Socio-demographic and major risk factors of study participants by age

Characteristics	Younger age (≤59)	Older age (≥60)	*P value*
	*N* (%)	*N* (%)	
Sex			
Male	117 (26.8)	90 (37.5)	0.001
Female	337 (74.2)	150 (62.5)	
Education			
Illiterate	154 (33.9)	142 (59.2)	<0.001
Primary school	172 (37.9)	58 (24.2)	
Secondary school & higher	128 (28.2)	40 (16.7)	
Income			
Low (<1000,000)	273 (65.0)	171 (75.7)	0.003
Acceptable	147 (35.0)	55 (24.36)	
Smoking			
Yes	33 (7.3)	26 (12.1)	0.07
No	421 (92.7)	214 (87.9)	
BMI			
< 25	79 (18.3)	41 (18.4)	0.705
25–29.9	178 (41.2)	83 (40.1)	
≥ 30	175 (40.5)	95 (41.5)	
Disease Duration (year)			
≤ 3	97 (21.5)	38 (15.8)	0.110
4–7	115 (25.4)	46 (19.2)	
≥ 7	240 (53.1)	156 (65)	
Hypertension			
No	196 (46.3)	87 (36.9)	0.019
Yes	227 (53.7)	149 (63.1)	
Total Cholesterol			
Desirable	365 (92.4)	198 (93.8)	0.317
≥ 240	30 (7.6)	13 (6.2)	
HbA1c			
< 7	143 (37)	77 (37)	0.99
≥ 7	244 (63)	131 (63)	
Complication			
No	249 (54.8)	103 (43.9)	0.003
Yes	205 (45.2)	137 (57.1)	

Table 2 shows the bivariate association between predictors and both outcomes. Females were more likely to have complications (*P* = 0.01), the frequency of complications was the lowest in the age group of ≤49, and the highest frequency was in the age group of 60+ (*P* = 0.001). The frequency of both outcomes was higher among illiterate people with type 2 diabetes (*P* = 0.003 to 0.01). The frequency of complications and poor glycemic control increased as the disease duration increased (*P* < 0.001, *P* = 0.003, respectively). Income, cholesterol level, hypertension, LDL and FBS were only associated with poor glycemic control not with complications (*P* < 0.001 to 0.002).

**Table 2:** Prevalence of complication and poor glycemic control among diabetic patients by socio-demographic and clinical factors, 2014–2015

Characteristics	Complications *N* (%)	*P* values	Poor glycemic control *N* (%)	*P value*
Sex				
Male	87 (42.0)	0.013	113 (64.2)	0.699
Female	255 (52.4)		262 (62.5)	
Age group				
≤ 49	59 (37.3)	0.001	88 (70.4)	0.118
50–59	146 (49.3)		156 (59.5)	
60+	137 (57.1)		131 (63.0)	
Education				
Illiterate	159 (53.7)	0.010	177 (69.7)	0.003
Primary school	117 (50.9)		109 (54.2)	
Secondary school & higher	66 (39.3)		89 (63.6)	
Income				
Low (<1000,000 Tomans^a^)	223 (50.2)	0.775	255 (70.2)	<0.001
Acceptable	99 (49.0)		92 (46.5)	
Duration of disease				
≤ 3 years	36 (26.7)	<0.001	65 (61.3)	0.003
4–7	66 (41.0)		72 (51.8)	
> 7 years	240 (60.6)		238 (68.0)	
Total Cholesterol				
Desirable	287 (51)	0.572	314 (60.4)	<0.001
≥ 240	20 (46.5)		36 (90.0)	
Smoking				
Yes	23 (39.0)	0.091	35 (62.5)	0.386
No	318 (50.5)		340 (68.6)	
BMI				
< 25	50 (41.7)	0.067	64 (62.1)	0.273
25–29.9	120 (46.0)		153 (68.0)	
≥ 30	144 (53.3)		141 (61.0)	
Hypertension				
Controlled	139 (49.1)	0.963	138 (55)	0.002
Uncontrolled (≥140/90)	184 (48.9)		217 (67.6)	
LDL				
Desirable	114 (54.0)	0.082	92 (47.9)	<0.001
≥ 100	60 (44.4)		85 (69.7)	

^a^1 US $ = 3200 Tomans

Table 3 shows the results of the univariate and multivariate logistic regression analyses. Compared to the age group of ≤49, the middle age group (50–59) and the older age group (60 years and older) were less likely to report poor glycemic control (OR fully adjusted = 0.49 95% CI: 0.28–0.86 and (OR = 0.44 95% CI: 0.24–0.80, respectively). The poor glycemic control was associated with income level, those with lower income were 2.6 times (95% CI: 1.68–3.90) more likely to have poor glycemic control compared with those having higher income. The same association holds for disease duration, hypercholesterolemia, high level of LDL and the hypertension (Table 3).

**Table 3:** Odds Ratio (95% confidence interval) of socio-demographic and clinical predictors with the presence of complications and poor glycemic control

Characteristics	Poor Glycemic Control			Presence of Complications		
	OR1 (95% CI)	OR2 (95% CI)	OR3 (95% CI)	OR1 (95% CI)	OR2 (95% CI)	OR3 (95% CI)
Age group						
≤ 49	Ref group	Ref group	Ref group	Ref group	Ref group	Ref group
50–59	0.62 (0.39–0.98)	0.57 (0.35–0.94)	0.49 (0.28–0.86)	1.63 (1.10–2.42)	1.53 (1.00–2.33)	1.13 (0.67–1.90)
≥ 60	0.72 (0.44–1.15)	0.51 (0.30–0.87)	0.44 (0.24–0.80)	2.23 (1.48– 3.37)	2.24 (1.41–3.54)	1.74 (1.00–3.02)
*P value*	0.119	0.039	0.020	0.001	0.003	0.076
Sex						
Male	Ref group	Ref group	Ref group	Ref group	Ref group	Ref group
Female	0.93 (0.65– 1.34)	1.25 (0.82–1.92)	0.73 (0.45–1.18)	1.52 (1.09–2.11)	1.57 (1.08–2.28)	1.15 (0.73 –1.82)
*P value*	0.699	0.304	0.198	0.013	0.019	0.560
Education						
Illiterate	1.32 (0.85 –2.04)	1.07 (0.63 –1.82)	1.20 (0.67 –2.17)	1.79 (1.22 –2.63)	1.39 (0.88–2.20)	1.96 (1.12–3.45)
Primary school	0.68 (0.44 – 1.06)	0.64 (0.39– 1.03)	0.75 (0.44–1.26)	1.60 (1.07 –2.40)	1.58 (1.03–2.42)	1.91 (1.14 –3.20)
Secondary school & higher	Ref group	Ref group	Ref group	Ref group	Ref group	Ref group
*P value*	0.003	0.037	0.152	0.01	0.114	0.027
Income						
Low <1000,000 Tomans	2.72 (1.90–3.89)	2.56 (1.75–3.76)	2.56 (1.68–3.90)	1.05 (0.75–1.46)	1.01 (0.76–1.44)	1.04 (0.68–1.60)
Acceptable	Ref group	Ref group	Ref group	Ref group	Ref group	Ref group
*P value*	<0.001	<0.001	<0.001	0.775	0.976	0.860
Duration of disease						
≤ 3 years	Ref group	Ref group	Ref group	Ref group	Ref group	Ref group
4–7	0.68 (0.41–1.13)	0.65 (0.37–1.13)	0.88 (0.48–1.63)	1.91 (1.17–3.13)	1.99 (1.16–3.42)	2.98 (1.54–5.76)
> 7 years	1.34 (0.85–2.10)	1.49 (0.91–2.45)	1.55 (0.90–2.65)	4.23 (2.75–6.51)	4.55 (2.82–7.33)	7.25 (4.00–13.12)
*P value*	0.004	0.001	0.043	<0.001	<0.001	<0.001
BMI						
< 25	Ref group	Ref group	Ref group	Ref group	Ref group	Ref group
25–29.9	1.30 (0.80–2.11)	1.28 (0.75–2.16)	1.92 (0.85–4.36)	1.19 (0.77–1.84)	1.20 (0.75–1.90)	1.42 (0.83–2.42)
≥ 30	0.96 (0.59–1.54)	089 (0.52–1.52)	1.05 (0.45–2.45)	1.60 (1.04–2.47)	1.42 (0.89–2.27)	1.66 (0.97–2.84)
*P value*	0.274	0.246	0.118	0.067	0.326	0.181
Total Cholesterol						
Desirable	Ref group	Ref group	Ref group	Ref group	Ref group	Ref group
≥ 240	5.90 (2/07 –16.84)	5.95 (1.76–20.14)	4.54 (1.29 –15.94)	0.84 (0.45–1/56)	0.93 (0.45–1.90)	1.30 (0.52–3.22)
*P value*	0.001	0.004	0.018	0.573	0.840	0.887
Hypertension						
Controlled	Ref group	Ref group	Ref group	Ref group	Ref group	Ref group
Uncontrolled	1.71 (1.22–2.40)	1.57 (1.09–2.26)	1.49 (1.00–2.22)	0.99 (0.73–1.35)	1.02 (0.73–1.41)	0.83 (0.56–1.23)
*P value*	0.002	0.016	0.052	0.963	0.929	0.354

OR1: Crude. OR2: Adjusted for age, gender, education and income. OR3: Adjusted for age, gender, education, income, disease duration, cholesterol, BMI, HTN

OR1: Crude. OR2: Adjusted for age, gender, education and income. OR3: Adjusted for age, gender, education, income, duration of disease, Cholesterol, BMI, HTN, and FBS

Relative to the younger age group, the middle age group (OR crude: 1.63 95% CI 1.10–2.42) and the older people with diabetes were more likely to report the presence of complication (OR crude = 2.23 95% CI 1.48 – 3.37). The associations remained statistically significant after adjustment for socio-demographic factors; however, it was attenuated after adjustment for all other variables. In fully adjusted model, the older people with diabetes in the age group of ≥60 were about 1.7 times more likely to have, at least, one complication (OR_adj_ =1.74 95% CI: 1.00–3.02). Among the middle age group, the association was attenuated in fully adjusted model and it shows a reduction and non-significant results.

Females were more likely to have complications than their male counterparts (OR crude = 1.52 95% CI: 1.09–2.11). Nevertheless, the association was attenuated in fully adjusted model and was no longer significant. Duration of disease was significantly associated with the presence of complications. In addition, people with duration of >7 years of disease were 6 times more likely to have complications (OR_adj_ = 5.98 95% CI: 2.35–15.22). Similarly, the same was the case with the middle-aged participants. Relative to those with higher level of education, people with diabetes in other education categories were about 2 times more likely to suffer from complications.

As can be seen in Table 4, compared to the age group of ≤49, complications were more likely to be present in the middle age group (OR = 1.62 95% CI: 1.09–2.42) and the same was true with the older age group (OR = 2.37 95% CI: 1.56–3.60). These associates remained significant even after the addition of other covariates such as FBS, BMI, cholesterol, duration of disease and hypertension. Thus, an independent role of age is suggested. However, there was not such a pattern for glycemic control. There was little reduction in the OR for the older age group in comparison with those in the age group of ≤49 which suggests a better glycemic control in these participants. It is important to note that these patterns remained the same with little attenuation after adjustment for different covariates.

**Table 4:** Changes in Odds Ratio (95% confidence interval) of age for complications and poor glycemic control with introduction of covariate

Outcome of Interest	Sex	FBS	Hypertension	Total cholesterol	BMI	Duration of diabetes
Glycemic control						
≤ 49	Ref group	Ref group	Ref group	Ref group	Ref group	Ref group
50–59	0.62 (0.39–0.98)	0.73 (0.44–1.19)	0.58 (0.36–0.93)	0.62 (0.39–1.00)	0.60 (0.37–0.96)	0.60 0.38–0.95)
60+	0.71 (0.44–1.15)	0.77 (0.46–1.29)	0.65 (0.40–1.07)	0.70 (0.43–1.16)	0.73 (0.44–1.20)	0.66 (0.40–1.07)
*P* value	0.12	0.44	0.08	0.15	0.10	0.087
Complications						
≤ 49	Ref group	Ref group	Ref group	Ref group	Ref group	Ref group
50–59	1.62 (1.09–2.42)	1.50 (1.00–2.25)	1.57 (1.04 – 2.38)	1.79 (1.16 –2.76)	1.42 (0.94–2.13)	1.52 (1.00 –2.29)
60+	2.37 (1.56–3.60)	2.23 (1.46 –3.40)	2.37 (1.54 – 3.66)	2.67 (1.69–4.20)	2.09 (1.36 –3.22)	2.03 (1.32–3.13)
*P* value	≤0.001	0.001	<0.001	<0.001	0.003	0.006

## Discussion

Of the participants of the study, 57.8% had poor glycemic, and 52.7% had, at least, one complication. Age, income, hypertension, duration of disease, hypercholesterolemia were independent predictors of poor glycemic control. However, only age, education and disease duration played an independent role in predicting complications.

Glycemic control was better among the middle and the older age groups in comparison with the younger age group where adjustment for other covariates had little effect on the association. Similarly, some studies reported better glycemic control among older age group [9, 10, 12, 18]. In contrast, complications increased as the age increased. This remained significant after adjustment for different covariates. There is evidence that diabetes complications, hospitalization and mortality are more prevalent among older people with diabetes [3–6]. Specifically speaking, results of our analysis were similar to the results of the Korean and Spanish studies i.e. in spite of a better glycemic control, with the older age group having the highest percentage of complications [5, 18].

In a Korean study, with a nationally representative sample of people with diabetes, age was an independent predictor for hospitalization (OR_adj_ = 1.97 95% CI = 1.28, 3.04) for the oldest group (ages 70–79) vs. youngest group (ages 40–49)) and a better glycemic control (OR_adj_ = 0.45 95% CI = 0.37, 0.56) for the oldest group vs. youngest group [5]. Barrot-de la Puente et al reported a better glycaemic control among older patients independent of disease duration, BMI and presence of a CVD [18].

There are some other studies which lend credence to the same results; the independent role of age in a better glycemic control [4, 10]. This might be partly explained by the increased attention paid to the medications of the older age in order to manage their blood glucose which suggests the need for improved glycemic management in younger patients.

Further, we examined the confounding or mediating role of other covariates in the association between the two outcomes of interest; complications and poor glycemic control. Simultaneous adjustment of sex and each covariate resulted in minor changes in the impact of age upon glycemic control. However, the association between age and the presence of complications changed a little after introducing FBS, but large changes in magnitude were observed for BMI and duration of disease (28 to 34% reduction in the OR between 60+ vs. ≤49 from 2.37 to 2.09 and 2.03, respectively). Adjustment for total cholesterol showed positive changes in the magnitude of the association for both the middle and older age groups (17 to 30%, respectively). In Korean study, only continuity of care provided a large change in the association between age and hospitalization [5]. This possibly could be explained with the difference in the population of the study and the management of diabetes in our clinics which indicates the need for a better management program for people with T2DM.

Despite useful findings of the present study, it has some limitations which need to be addressed, as they might provide an agenda for future studies. The main limitation of the present study was applying convenience sampling method which yields non-representative study participation. However, incomplete clinic records; personal identification, address change, death or migration were the main reasons for using this method. In addition, the majority of the study participations were women. Therefore, gender differences could not be adequately studied in this analysis. As this study was conducted in governmental clinics, the results might differ from private sectors reflecting different diabetes care and management. Finally, using a more rigorous sampling method and inclusion of participants from both governmental and private sectors are recommended by the authors in order to improve the generalizability of findings in future studies.

## Conclusion

According to the results of this study, the older diabetic patients had a higher percentage of complications despite displaying a better glycemic control. This might highlight the complexity of management of diabetes among older patients. The influential factors were variably associated with the two diabetes-related outcomes, providing more comprehensive risk profiles for glycemic control. Functional capacity and comorbidities may explain the increased complication among older diabetic patients despite showing a better glycemic control and suggests a wider geriatric evaluation.

## Additional file

Additional file 1: Project questioner (DOCX 70 kb)

## Abbreviations

BMI: Body mass index
CI: Confidence intervals
HDL: High dense lipoprotein
LDL: Low dense lipoprotein
OR: Odds ratio
ORadj: Adjusted odds ratio
ORcrude: Crude odds ratio
T2DM: Type 2 diabetes mellitus

## Footnotes

1 1 US dollar equals to 32,00 Iranian Tomans.
