# Supplementary material for: Age differences in diabetes-related complications and glycemic control
Source: BMC Endocr Disord. 2017 May 4;17:25. doi: 10.1186/s12902-017-0175-5 (PMC5418847; doi:10.1186/s12902-017-0175-5)
Supplement: Additional file 1: — Project questioner (DOCX 70 kb) [file 12902_2017_175_MOESM1_ESM.docx]

**Demographic and health care information of diabetic patients**

**This information is related to the Diabetic project No: TBZMED.REC.1392.207 AND, TBZMED.REC.1394.55**

**Patient project Number:** ………………………………

| **Tel:**  Lane:  Mob: | **Last name:** | **First name:** |
| --- | --- | --- |
| **Sex:** Male Female | | **Date of Birth:** (DD/MM/YY) |
| **Height(cm):** | | **Wight (kg):** |
| **Marital status:** Widowed Divorced Married Single | | |
| **Job:**  Employed Self-employed  Housekeeper Retired  Other specify ……………………….. | | **Income (Toman):**  < 500,000  500,000-1000,000  > 1000,000 |
| **Education:** Illiterate Primary school Secondary school & higher | | |
| **Health Insurance:** Yes No | | |
| **FBS:** | | **HbA1C:** |
| Dia BP: Sys BP: **Blood pressure:** | | |
| **Lipid profile:** Total cholesterol: HDL: LDL: | | |
| **Micro-albuminuria:** Yes No | | |
| **ACEI therapy:** Yes No | | |
| List of Medications:  1- 2-  3- 4-  5- 6-  7- 8- | | **Therapeutic methods:**  Diet only  Oral medication  Insulin Injection  Oral medication + Insulin Injection |
| **Comorbidity Diseases:**  Depression Hypertension Kidney disease Cancer  Cardio vascular disease Other Specify ……………………………………………… | | |
| **Presence of Complications** No Yes  If Yes Please specify: ………………………………………………………………………………. | | |
| **Duration Of disease (month) ………….** | | |
| **Familial history of Diabetes:** Yes No | | |

**Quality of life Questionnaire**

| **Very Good** | **Good** | **Neither poor nor good** | **Poor** | **Very poor** | **Questions** | **No.** |
| --- | --- | --- | --- | --- | --- | --- |
|  |  |  |  |  | How would you rate your quality of life? | 1 |
|  |  |  |  |  | How satisfied are you with your health | 2 |
|  |  |  |  |  | To what extent do you feel that physical pain prevents you from doing what you need to do? | 3 |
|  |  |  |  |  | How much do you need any medical treatment to function in your daily life? | 4 |
|  |  |  |  |  | How much do you enjoy life? | 5 |
|  |  |  |  |  | To what extent do you feel your life to be meaningful | 6 |
|  |  |  |  |  | How well are you able to concentrate? | 7 |
|  |  |  |  |  | How safe do you feel in your daily life | 8 |
|  |  |  |  |  | How healthy is your physical environment? | 9 |
|  |  |  |  |  | Do you have enough energy for everyday life? | 10 |
|  |  |  |  |  | Are you able to accept your bodily appearance? | 11 |
|  |  |  |  |  | Have you enough money to meet your needs? | 12 |
|  |  |  |  |  | How available to you is the information that you need in your day-to-day life? | 13 |
|  |  |  |  |  | To what extent do you have the opportunity for leisure activities? | 14 |
|  |  |  |  |  | How well are you able to get around | 15 |
|  |  |  |  |  | How satisfied are you with your sleep? | 16 |
|  |  |  |  |  | How satisfied are you with your ability to perform your daily living activities? | 17 |
|  |  |  |  |  | How satisfied are you with your capacity for work? | 18 |
|  |  |  |  |  | How satisfied are you with yourself | 19 |
|  |  |  |  |  | How satisfied are you with your personal relationships? | 20 |
|  |  |  |  |  | How satisfied are you with your sex life | 21 |
|  |  |  |  |  | How satisfied are you with the support you get from your friends? | 22 |
|  |  |  |  |  | How satisfied are you with the conditions of your living place? | 23 |
|  |  |  |  |  | How satisfied are you with your access to health services? | 24 |
|  |  |  |  |  | How satisfied are you with your transport? | 25 |
|  |  |  |  |  | How often do you have negative feelings such as blue mood, despair, anxiety, depression? | 26 |

**Functional disability**

The following items are activities you might do during a typical day. Does your health limit you in these activities?

| **No, not limited at all** | **Yes, limited a little** | **Yes, limited a lot** | **Questions** | **No.** |
| --- | --- | --- | --- | --- |
|  |  |  | Vigorous activities, such as running, lifting heavy objects, participating in strenuous sports. | 1 |
|  |  |  | Moderate activities, such as moving a table, pushing a vacuum cleaner, bowling, or playing golf. | 2 |
|  |  |  | Lifting or carrying groceries. | 3 |
|  |  |  | Climbing several ﬂights of stairs | 4 |
|  |  |  | Climbing 1 ﬂight of stairs | 5 |
|  |  |  | Bending, kneeling, or stooping. | 6 |
|  |  |  | Walking more than a mile. | 7 |
|  |  |  | Walking several blocks. | 8 |
|  |  |  | Walking. one block | 9 |
|  |  |  | Bathing or dressing yourself. | 10 |

**Kessler Psychological Distress Scale (K10)**

| **In the last four weeks, about how often did you feel?** | | | | | | |
| --- | --- | --- | --- | --- | --- | --- |
| **Items** | | **All of the time** | **Most of the time** | **Some of the time** | **A little of the time** | **None of the time** |
| 1 | Tired out for no good reasons? |  |  |  |  |  |
| 2 | Nervous? |  |  |  |  |  |
| 3 | So nervous that nothing could calm you down? |  |  |  |  |  |
| 4 | Hopeless? |  |  |  |  |  |
| 5 | Restless or fidgety? |  |  |  |  |  |
| 6 | So restless that you could not sit still? |  |  |  |  |  |
| 7 | Depressed? |  |  |  |  |  |
| 8 | That everything was an effort? |  |  |  |  |  |
| 9 | So sad that nothing could cheer you up? |  |  |  |  |  |
| 10 | Worthless? |  |  |  |  |  |

**Interviewer’s name and signature:**

**Date:**
